# miR-188-3p targets skeletal endothelium coupling of angiogenesis and osteogenesis during ageing

**DOI:** 10.1038/s41419-022-04902-w

**Published:** 2022-05-25

**Authors:** Wen-Zhen He, Mi Yang, Yangzi Jiang, Chen He, Yu-Chen Sun, Ling Liu, Mei Huang, Yu-Rui Jiao, Kai-Xuan Chen, Jing Hou, Min Huang, Yi-Li Xu, Xu Feng, Ya Liu, Qi Guo, Hui Peng, Yan Huang, Tian Su, Ye Xiao, Yusheng Li, Chao Zeng, Guanghua Lei, Xiang-Hang Luo, Chang-Jun Li

**Affiliations:** 1 Department of Endocrinology, Endocrinology Research Center, Xiangya Hospital of Central South University, Changsha, 410008 Hunan China; 2 Institute for Tissue Engineering and Regenerative Medicine (iTERM), School of Biomedical Sciences, The Chinese University of Hong Kong, Hong Kong, Hong Kong; 3 Department of Orthopaedics, Xiangya Hospital of Central South University, Changsha, 410008 Hunan China; 4 Hunan Key Laboratory of Joint Degeneration and Injury, Changsha, 410008 Hunan China; 5 National Clinical Research Center for Geriatric Disorders, Xiangya Hospital, Central South University, Changsha, 410008 Hunan China; 6 Key Laboratory of Organ Injury, Aging and Regenerative Medicine of Hunan Province, Changsha, 410008 Hunan China

**Keywords:** Osteoporosis, Senescence

## Abstract

A specific bone capillary subtype, namely type H vessels, with high expression of CD31 and endomucin, was shown to couple angiogenesis and osteogenesis recently. The number of type H vessels in bone tissue declines with age, and the underlying mechanism for this reduction is unclear. Here, we report that microRNA-188-3p (miR-188-3p) involves this process. miRNA-188-3p expression is upregulated in skeletal endothelium and negatively regulates the formation of type H vessels during ageing. Mice with depletion of miR-188 showed an alleviated age-related decline in type H vessels. In contrast, endothelial-specific overexpression of miR-188-3p reduced the number of type H vessels, leading to decreased bone mass and delayed bone regeneration. Mechanistically, we found that miR-188 inhibits type H vessel formation by directly targeting integrin β3 in endothelial cells. Our findings indicate that miR-188-3p is a key regulator of type H vessel formation and may be a potential therapeutic target for preventing bone loss and accelerating bone regeneration.

## Introduction

Blood vessels present in bone tissue and actively participate in bone development, homeostasis and regeneration [1, 2]. A specific capillary subtype in bone tissues was recently characterized and named type H vessels, characterized by the high expression of endothelial markers CD31 and endomucin (Emcn) with regional and functional specificity [3]. Type H vessels are strongly coupled with angiogenesis and osteogenesis and are located in the metaphysis and endosteum of the diaphysis [3]. Type H vessels create a special microenvironment that can support osteoprogenitor function and give rise to osteoblasts and osteocytes [3, 4]. Promoting type H vessel formation could ameliorate bone loss in the ageing-, estrogen deficiency- and glucocorticoid-induced osteoporosis mice models [4–6]. Moreover, type H vessels digest the cartilage matrix and mediate the directional bone elongation, which is essential for bone development [7]. Several pathways and factors have been shown to regulate type H vessel formation, such as the *Notch* pathway, *Hif-1α*, *PDGF-BB*, and *Slit3* [3–5, 8–10]. MicroRNAs (miRNAs) have also been reported to play central roles in regulating type H vessel formation [11–13]. Yang et al. found that *miR-497–195* cluster expression decreases with ageing, leading to a decline in type H vessels [11]. However, restoration of the *miR-497–195* cluster level in endothelial cells (ECs) could only partially rescue the loss of type H vessels with age [11], indicating that other factors may also be involved in regulating the type H vessel decline in ageing.

Type H vessels were also reported to be associated with bone regeneration [4, 6]. Several type H vessel anabolic reagents, including ophiopogonin D, a natural compound, and exogenous SLIT3 can accelerate bone fracture healing and repair. The regenerative ability of bone tissue declined with ageing [14]. It is unclear whether the age-related type H vessel decline contributes to impaired bone regeneration in ageing. If so, can preservation of type H vessels attenuate bone fracture healing impairment in ageing?

In our previous study, we identified miR-188 as a key regulator of age-related bone marrow mesenchymal stem cell (BMSC) lineage fate switching between osteoblasts and adipocytes during skeletal ageing [15]. miR-188 has been reported to be involved in multiple age-related diseases, such as ageing-associated metabolic dysfunction and Alzheimer’s disease [16, 17], and cellular senescence in various types of cells [18, 19]. As type H vessels are tightly coupled with osteogenesis in spatial and molecular manners, it is possible that miR-188 could also regulate this type of skeletal vessels during ageing. We hypothesized here that miR-188 is one of the mediator of type H vessels, and involves in bone fracture healing impairment. There are thus needs to further investigate the overall biological effect of miR-188 in ageing, and the therapeutic effects of miR-188 on age-related diseases and pathological processes.

Integrins, as transmembrane proteins, have been identified as the main receptors for the extracellular matrix for cell adhesion [20]. There are 24 integrin heterodimers encoded by 18 α integrin subunits and eight β integrin subunit genes [21]. These molecules are known to regulate cell behavior, including survival, proliferation, differentiation, and motility, by mediating the translation of spatial extracellular signals [22–24]. A previous study showed that transcripts for integrins were enriched in type H ECs, and one member of the integrin family, integrin β1, regulates the morphological and functional properties of the type H endothelium [25]. Integrin β3, as an integrin subunit, participates in the formation of αVβ3, which is an important membrane receptor that senses extracellular signals and mediates angiogenesis [26–28].

Here, we found that mice with knockout of miR-188 showed an alleviated age-related decline of type H vessels, suggesting that miR-188 plays an important role in the formation and preservation of type H vessels. We then found that senescent ECs express higher levels of miR-188-3p, which lead to impaired angiogenesis. Mice with overexpression of miR-188-3p in ECs showed fewer type H vessels, impaired bone formation and delayed bone regeneration. We then identified integrin β3 (*ITGβ3*) as the direct target of miR-188-3p, which could possibly mediate the formation of type H vessels. Taken together, our findings reveal that miR-188-3p is a key regulator of type H vessels and may be a potential therapeutic target for preventing bone loss and accelerating bone regeneration.

## Results

### Mice with miR-188 knockout showed an alleviated decline in type H vessels with age

**Fig. 1:**
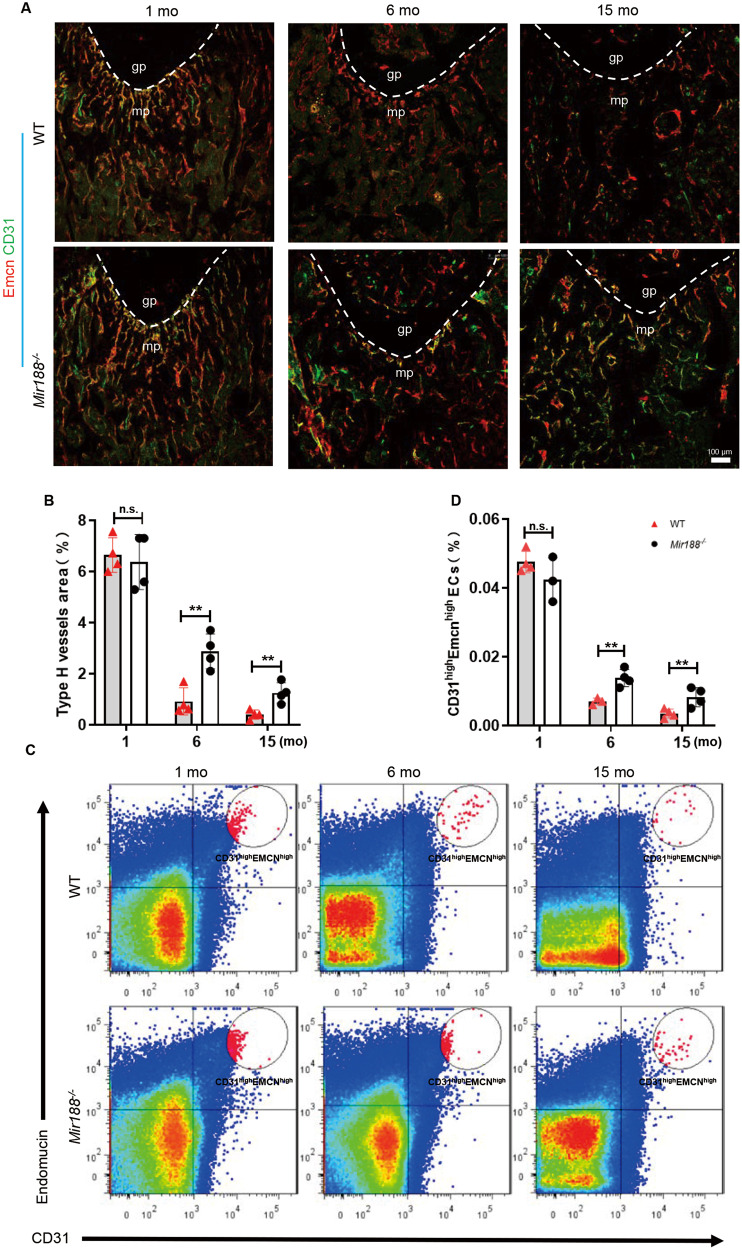
miR-188 knockout mice showed an alleviated decline in type H vessels with age. **A** Representative images of coimmunostaining of Emcn (red) and CD31 (green) in femur sections from 1-month-, 6-month-, and 15-month-old WT and miR-188 knockout (*Mir188*^*−/−*^) mice. Scale bar = 100 µm. **B** Quantification of type H vessels in femur sections from 1-month-, 6-month-, and 15-month-old WT and *Mir188*^*−/−*^ mice. *n* = 4 mice in each group. **C** Representative images of Flow-cytometry analysis dot plot from the long bones of 1-month-, 6-month-, and 15-month-old WT and *Mir188*^*−/−*^ mice. **D** Quantitative flow-cytometry analysis of CD31^high^ and Emcn^high^ cells from the long bones of 1-month-, 6-month-, and 15-month-old WT and *Mir188*^*−/−*^ mice. *n* = 3 (1-month *Mir188*^*−/−*^ mice and 6-month WT mice), *n* = 4 (1-month WT mice, 6-month *Mir188*^*−/−*^ mice, 15-month WT mice and 15-month *Mir188*^*−/−*^ mice). gp growth plate, mp metaphysis, mo month. Data are shown as the mean ± SD. n.s. no significance; **P < 0.01 by Student’s *t* test.

The number of type H vessels were examined in mice by coimmunostaining for CD31 and Emcn, and a strong reduction in the number of type H vessels during ageing (Fig. S1A, B) was observed, consistent with a previous study [3]. To investigate whether miR-188 involves in age-related type H vessel regulation, mice with miR-188 knockout (*Mir188*^−*/−*^) were generated, and the type H vessels in bone were analysed. Coimmunostaining of CD31 and Emcn showed that the amount of CD31^high^Emcn^high^ endothelium in the femora was not significantly different between the *Mir188*^−*/−*^ mice and the wild-type (WT) mice at 1 month of age (Fig. 1A, B). However, the amount of CD31^high^Emcn^high^ endothelium was substantially higher in both the 6- and 15-month-old *Mir188*^−*/−*^ mice than in the age-matched WT mice (Fig. 1A, B). To further confirm above results, we performed flow cytometric analysis and found that the number of CD31^high^Emcn^high^ ECs in the bone marrow of the 6- and 15-month-old *Mir188*^−*/−*^ mice was higher than that of the age-matched WT mice, while no difference in the CD31^high^Emcn^high^ endothelial cell number between the 1-month-old *Mir188*^−*/−*^ mice and the WT control mice was observed (Fig. 1C, D).

The above data indicate that miR-188 may play a vital role in the type H vessel decline during ageing.

### miR-188-3p expression increased during senescence and inhibited angiogenesis

**Fig. 2:**
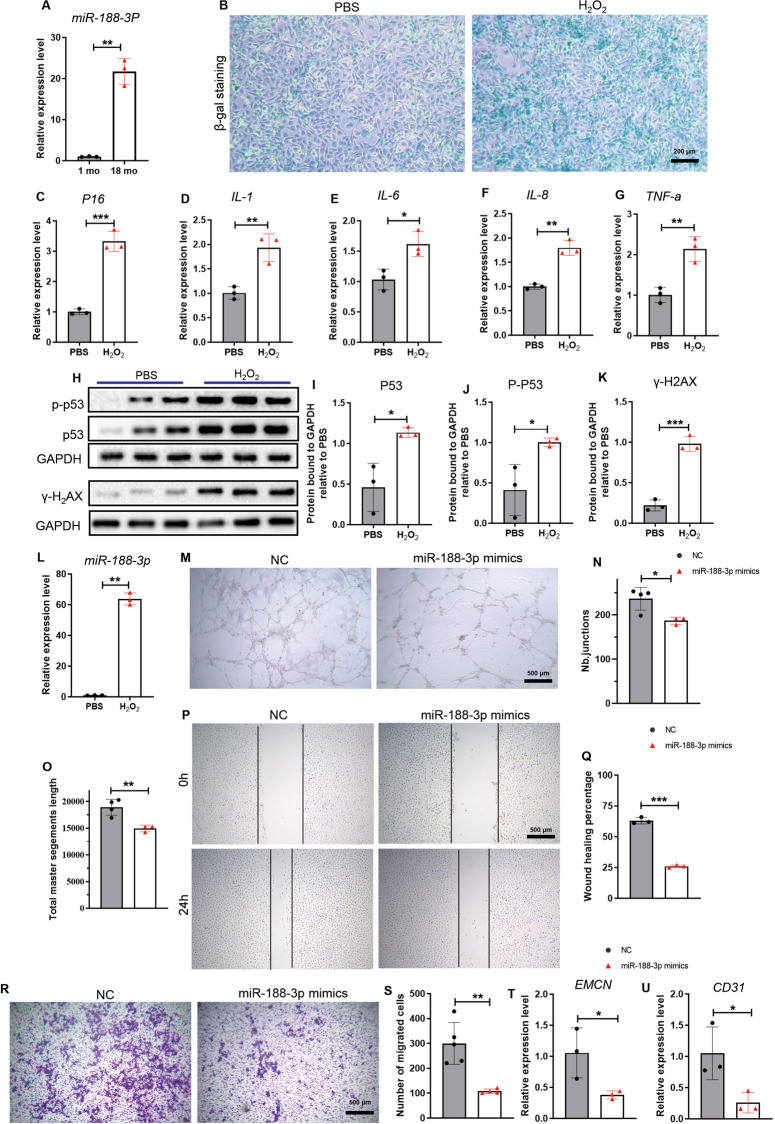
miR-188-3p expression increased during senescence and inhibited angiogenesis in vitro. **A** The expression of miR-188-3p in primary endothelial progenitor cells from the age-indicated male C57BL/6 mice. *n* = 3 in each group. **B** Representative images of β-gal staining from HMEC-1 treated with PBS or H_2_O_2_. Scale bar = 200 µm. **C**–**G** The relative expression of Ink4a-p16 and SASP factors (IL-1, IL-6, IL-8, TNF-a) in the HMEC-1 cells treated with PBS or H_2_O_2_. *n* = 3 in each group. **H**–**K** Images of western blotting (**H**) and quantitative analysis of P53, P-P53, γ-H2AX protein expression levels (**I**–**K**) in the HMEC-1 cells treated with PBS or H_2_O_2_. *n* = 3 in each group. **L** The expression of miR-188-3p in HMEC-1 treated with PBS or H_2_O_2_. *n* = 3 in each group. **M** Representative images of tube formation assays from the groups transfected with miR-188-3p mimics or miRNA-NC. Scale bar = 500 μm. **N**, **O** Quantification of tube junction numbers (**N**) and total master segment length (**O**) in the tube formation assays of the indicated groups. *n* = 3 miR-188-3p mimic-treated groups, *n* = 4 miRNA-NC-treated groups. **P**, **Q** Representative images (**P**) and wound healing percentage (**Q**) of the endothelial cell line transfected with miR-188-3p or miRNA-NC in the wound healing assays. Scale bar = 500 μm. *n* = 3 in each group. **R**, **S** Representative images (**R**) and quantitative analysis (**S**) of the migrated cells from a Transwell migration assay. Scale bar = 500 μm. *n* = 4 miR-188-3p-treated group, *n* = 5 miRNA-NC-treated group. **T**, **U** The relative expression of *Emcn* (**T**) and *CD31* (**U**) in the HMEC-1 cells transfected with miR-188-3p mimics or miRNA-NC. *n* = 3 in each group. NC: treated with miRNA-NC, miR-188-3p mimics: treated with miR-188-3p mimics. Data are shown as the mean ± SD. n.s. no significance; *P < 0.05; **P < 0.01; ***P < 0.001 by two-tailed Student’s *t* test or Welch’s *t t*est.

Next, we tested the expression levels of miR-188 in senescent endothelial cells. The gene expression analysis for miR-188-3p and miR-188-5p in primary ECs isolated from young (1 mo) and older (18 mo) mice were obtained, and we found that the expression of both miR-188-3p and miR-188-5p was increased in ECs from the older mice compared to those from the young mice (Figs. 2A, S2A). To investigate the role of miR-188-3p and miR-188-5p in endothelial cell senescence, we used hydrogen peroxide (H_2_O_2_) induced endothelial cell lines (HMEC-1) experimental senescence model to represent the endothelial cell ageing process in vitro [29, 30]. The positive staining of senescence-associated beta-galactosidase (SA-β-gal), higher expression of *ink4a-p16* and SASP factors, accumulated γ-H2AX foci and phosphorylated p53 [31–34] indicated that senescent endothelial cell phenotypes were successfully induced by H_2_O_2_ (Fig. 2B–K), and we found that the expression of both miR-188-3p and miR-188-5p was increased in senescent HMEC-1 cells (Figs. S2B, 2L).

Since both miR-188-3p and miR-188-5p was elevated in senescent ECs, we next examined which miRNA subtype plays a major role in regulating angiogenesis. Overexpression of miR-188-3p strongly impaired tube formation in HMEC-1 cells, while miR-188-5p overexpression had no such effect (Figs. S2C–G, 2M–O).

Endothelial cell migration is essential for angiogenesis [34]. ECs overexpressing miR-188-3p showed impaired migration, as determined by wound healing assays and Transwell assays (Fig. 2P–S). Moreover, overexpression of miR-188-3p downregulated the expression of *Emcn* and *Pecam1* (gene encoding CD31) in ECs (Fig. 2T–U), suggested possibly inhibited angiogenesis. Next, we tested the function of miR-188-3p in regulating endothelial cell senescence by overexpression miR-188-3p in HMEC-1, and no dramatic difference was observed from the expression levels of *P16*, SA-β-gal staining, SASP factors, γ-H2AX foci, and phosphorylated p53, compared with the miR-NC control cells (Fig. S3A–J).

Our previous study found that miRNA-188-5p regulates the age-related switch of BMSCs between osteoblast and adipocyte differentiation [15], which made us wonder whether miR-188-3p plays a role in regulating BMSC differentiation. We then transfected BMSCs with miR-188-3p and then examined the osteogenic and adipogenic differentiation. Surprisingly, the expression of the osteogenic genes *Runx2*, *Alp*, and *Sp7*, as well as mineralization, was not significantly changed in the miR-188-3p-transfected BMSCs compared to the miR-NC-treated BMSCs, as shown by qPCR analysis and Alizarin Red staining (Fig. S4A–D). In addition, qPCR analysis of adipogenic genes, such as *Fabp4* and *Pparg*, and Oil red O staining showed no significant difference between the miR-188-3p mimic- and miR-NC-treated groups (Fig. S4E–G). Moreover, BMSC migration was not affected by miR-188-3p overexpression (Fig. S4H, I).

These data indicated that miR-188-3p negatively regulated angiogenesis without affecting osteogenesis, adipogenesis or migration of BMSCs.

### Mice with endothelial cell-specific miR-188-3p overexpression showed reduced type H vessels and bone mass

**Fig. 3:**
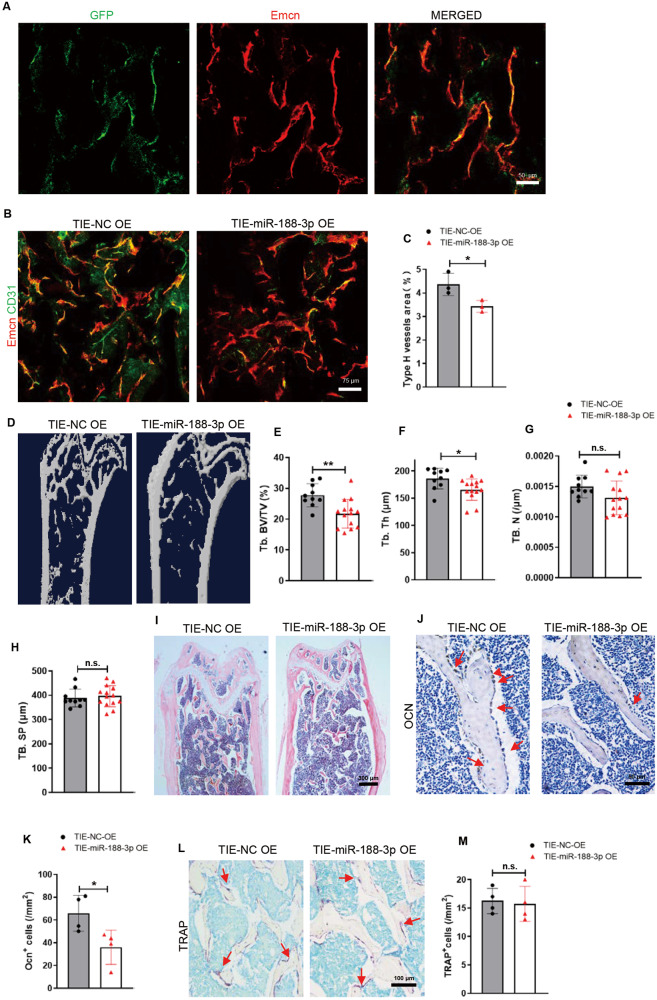
Mice with endothelial cell-specific miR-188-3p overexpression showed reduced type H vessels and bone mass. **A** Representative images of Emcn (red) and GFP (green) immunostaining. Scale bar = 50 μm. **B** Representative images of Emcn (red) and CD31 (green) immunostaining of femur sections from the indicated groups. Scale bar = 75 μm. **C** Quantification of the type H vessels in femur sections. *n* = 3 in each group. **D**–**H** Representative microcomputed tomography (μCT) images (**D**) and quantitative μCT analysis (**E**–**H**) of trabecular bone microarchitecture in femora from the 3-month-old mice with endothelial cell-specific miR-188-3p or miRNA-NC overexpression. *n* = 10 mice with endothelial cell-specific miRNA-NC overexpression, *n* = 14 mice with endothelial cell-specific miR-188-3p overexpression. **I** Representative images of haematoxylin-eosin staining in femora of the 3-month-old mice with endothelial cell-specific miR-188-3p or miRNA-NC overexpression. Scale bar = 500 μm. **J**, **K** Representative images (**J**) and quantitative analysis (**K**) of osteocalcin immunohistochemical staining from the 3-month-old mice with endothelial cell-specific miR-188-3p or miRNA-NC overexpression. Scale bar = 50 μm. *n* = 4 in each group. **L**, **M** Representative images (**L**) and quantitative analysis (**M**) of TRAP staining from the 3-month-old mice with endothelial cell-specific miR-188-3p or miRNA-NC overexpression. Scale bar = 100 μm. *n* = 4 in each group. TIE-NC OE overexpression of miRNA-NC in endothelial cells, TIE-3P OE overexpression of miR-188-3p in endothelial cells. Data are shown as the mean ± SD. n.s. no significance; *P < 0.05; **P < 0.001 by two-tailed Student’s *t* test.

To study the role of miR-188-3p in type H vessels, we took advantage of adeno-associated virus (AAV) technology with endothelial cell promoter (TIE)-customized AAV [35]. AAV-TIE-GFP was used to detect the specificity and efficiency of the customized AAV. The majority of the GFP signal colocalized with the Emcn-red signal, indicating that customized AAV-mediated miR-188-3p overexpression specifically occurred in ECs (Fig. 3A). Then, we administered AAV-TIE-miR-188-3p to mice (young mice, 3-month-old) *via* an intramedullary injection in the femora to overexpress miR-188-3p specifically in bone marrow ECs. At 5 weeks post-injection, we euthanized these mice and harvested the femoral tissue followed by immunofluorescence staining of CD31 and Emcn. In contrast to those in the AAV-TIE-miR-NC-treated control mice, fewer types of type H endothelium were detected in the AAV-TIE-miR-188-3p-treated mice (Fig. 3B, C). Previous studies reported that type H vessels prevent bone loss by coupling osteogenesis [3, 4, 8, 9]. We next measured the bone mass of the AAV-TIE-miR-188-3p-treated mice. Micro-CT analysis revealed that the bone volume fraction and trabecular thickness were lower in the femora of the AAV-TIE-miR-188-3p-treated mice than in the femora of the control mice (Fig. 3D–H). Haematoxylin and eosin (HE) staining further showed that the miR-188-3p-overexpressing mice had significantly lower trabecular bone levels than their controls (Fig. 3I). The number of osteocalcin-positive osteoblasts was decreased, while the number of TRAP-positive osteoclasts was not affected in the AAV-TIE-miR-188-3p-treated mice (Fig. 3J–M).

Thus, overexpression of miR-188-3p in ECs reduced the number of type H ECs accompanied by decreased osteoblastic bone formation and bone mass.

### Mice with endothelial cell-specific miR-188-3p overexpression showed reduced type H vessels and delayed bone regeneration during repair of bone damage

**Fig. 4:**
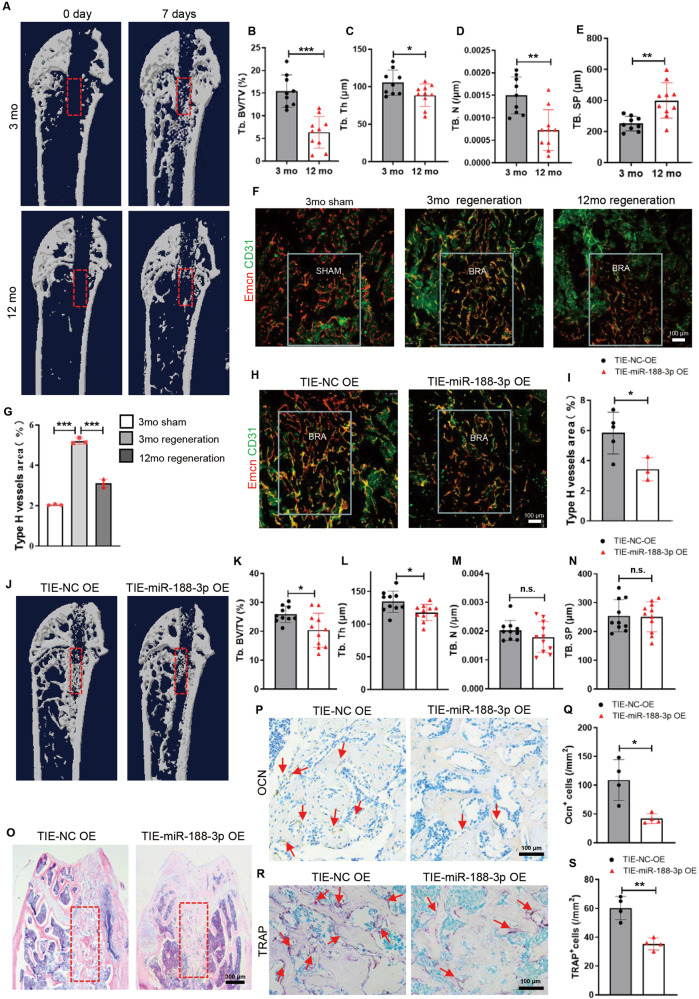
Mice with endothelial cell-specific miR-188-3p overexpression showed reduced type H vessels and delayed bone regeneration during bone damage repair. **A** Representative μCT images of trabecular bone microarchitecture in the bone regeneration area from 3-month-old and 12-month-old mice 0 days and 7 days after surgery. **B**–**E** Quantification of the trabecular bone microarchitecture in the femora of the bone generation area from 3-month-old and 12-month-old mice. *n* = 9 (3-month-old mice), *n* = 10 (12-month-old mice). **F**, **G** Representative images (**F**) and quantitative analysis (**G**) of Emcn (red) and CD31 (green) immunostaining of the bone regeneration area in femur sections from the indicated mice. Scale bar = 100 μm. *n* = 3 in each group. **H**, **I** Representative images (**H**) and quantitative analysis (**I**) of Emcn (red) and CD31 (green) immunostaining of the bone regeneration area in femur sections from the 3-month-old mice with endothelial cell-specific miR-188-3p or miRNA-NC overexpression. Scale bar = 100 μm. *n* = 5 mice with endothelial cell-specific miRNA-NC overexpression, *n* = 3 mice with endothelial cell-specific miR-188-3p overexpression. **J**–**N** Representative μCT images (**J**) and quantification (**K**–**N**) of trabecular bone microarchitecture in the bone generation area from the 3-month-old mice with endothelial cell-specific miR-188-3p or miRNA-NC overexpression. *n* = 10 mice with endothelial cell-specific miRNA-NC overexpression, *n* = 11 mice with endothelial cell-specific miR-188-3p overexpression. **O** Representative images of haematoxylin-eosin staining in the bone generation area of the 3-month-old mice with endothelial cell-specific miR-188-3p or miRNA-NC overexpression. Scale bar = 500 μm. **P**, **Q** Representative images (**P**) and quantitative analysis (**Q**) of osteocalcin immunohistochemical staining in the bone generation area from the 3-month-old mice with endothelial cell-specific miR-188-3p or miRNA-NC overexpression. Scale bar = 100 μm. *n* = 4 in each group. **R**, **S** Representative images (**R**) and quantitative analysis (**S**) of TRAP staining in the bone generation area from the 3-month-old mice with endothelial cell-specific miR-188-3p or miRNA-NC overexpression. Scale bar = 100 μm. *n* = 4 in each group. TIE-NC OE overexpression of miRNA-NC in endothelial cells, TIE-3P OE overexpression of miR-188-3p in endothelial cells. BRA bone regeneration, mo month. Data are shown as the mean ± SD. n.s. no significance; *P < 0.05; **P < 0.01; ***P < 0.001 by one-way ANOVA in (**G**), and two-tailed Student’s *t* test or Welch’s *t* test in the rest panels.

Next, we tested whether endothelium miR-188-3p overexpression reduced type H vessels are coupled with impaired bone regeneration during ageing. We generated a bone defect model by surgical ablation of the trabecular bone in young (3-month-old) and older (12-month-old) mice. Micro-CT analysis revealed that the bone volume regenerated in the defect area was increased in both young and old mice at 1 week after surgery. However, the bone volume in the regeneration area of the old mice was lower than that of the young mice (Fig. 4A–E). Moreover, we found that type H vessels increased at the bone regeneration area in both young and old mice, while the amount of type H vessels in the old mice was much lower than that in the young mice at 1-week post-surgery (Fig. 4F, G), which indicates that impaired bone regeneration was associated with reduced type H vessels in old mice.

To further assess whether the miR-188-3p-induced type H vessel reduction also impairs bone regeneration, we created a bone defect in mice with endothelial cell-specific miR-188-3p overexpression. As expected, overexpression of miR-188-3p in ECs reduced the CD31^high^Emcn^high^ endothelium in the new bone area (Fig. 4H, I). Then, we measured the bone regeneration phenotype. Micro-CT analysis and HE staining showed a significantly lower bone mass in the defect area in the mice with endothelial cell-specific miR-188-3p overexpression compared with that in the control group (Fig. 4J–O). The number of osteocalcin (Ocn)-positive mature osteoblasts and TRAP-positive mature osteoclasts was decreased in the bone regenerating area of the endothelial cell-specific miR-188-3p-overexpressing mice (Fig. 4P–S), which indicated impaired bone regeneration in the endothelial cell-specific miR-188-3p-overexpressing mice.

These data suggested that the weakened ability of bone regeneration with age is closely correlated with the decline in type H vessels at the injury site, and endothelial cell-specific overexpression of miR-188-3p reduces type H vessels and delays bone regeneration.

### Mice with knockout miR-188 showed more type H vessels and accelerated bone regeneration during repair of bone damage

**Fig. 5:**
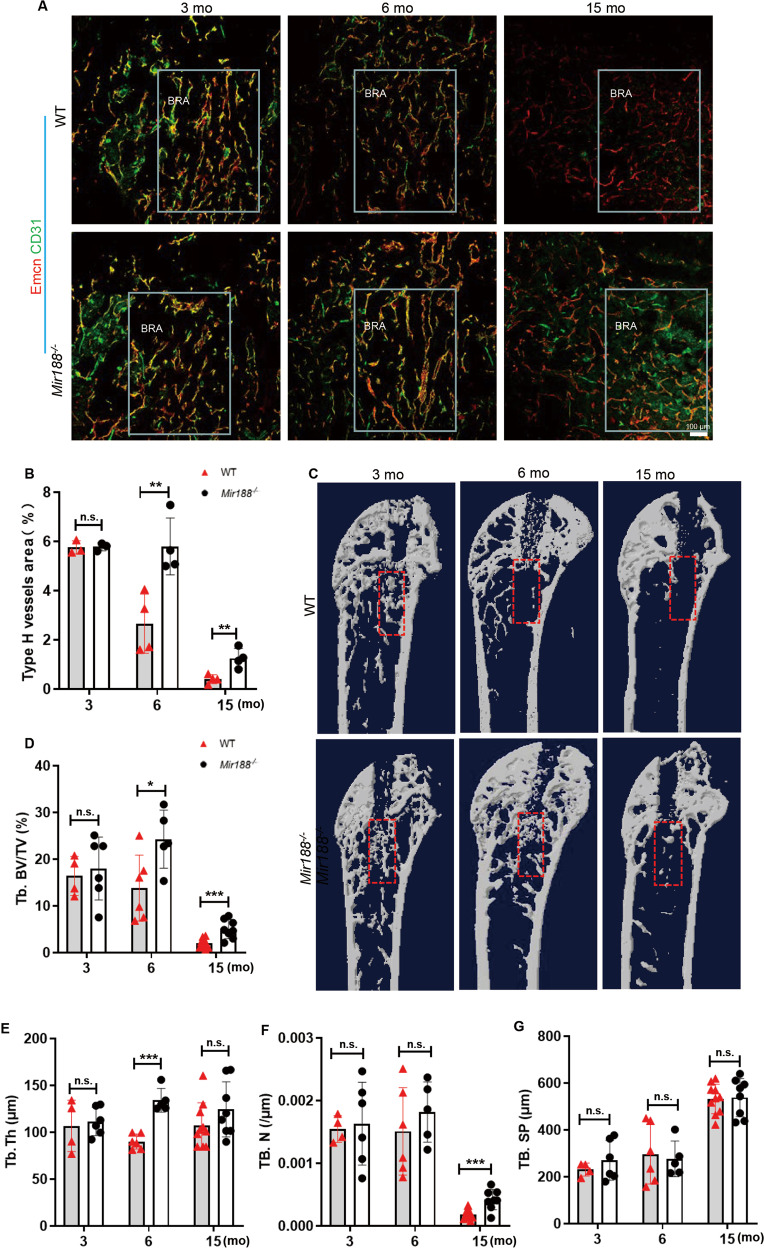
Mice with knockout miR-188 showed more type H vessels and accelerated bone regeneration during bone damage repair. **A** Representative images of Emcn (red) and CD31 (green) immunostaining of the bone regeneration area in femur sections from the indicated mice. Scale bar = 100 μm. **B** Quantitative analysis of type H vessels in the bone regeneration area of femora from the indicated mice. *n* = 3 (3-month-old WT or *miR188*^*−/−*^ mice), *n* = 4 (6-month-old and 15-month-old WT or *miR188*^*−/−*^ mice). **C** Representative μCT images of trabecular bone microarchitecture in the bone regeneration area from 3-month-, 6-month-, and 15-month-old WT or *miR188*^*−/−*^ mice 0 days and 7 days after surgery. **D**–**G** Quantitative analysis of trabecular bone microarchitecture in the bone generation area from the indicated mice. *n* = 4 (3-month WT mice), *n* = 6 (3-month *miR188*^*−/−*^ mice and 6-month WT mice), *n* = 5 (6-month *miR188*^*−/−*^ mice), *n* = 10 (15-month WT mice), *n* = 8 (15-month *miR188*^*−/−*^ mice). BRA: bone regeneration; mo: month. Data are shown as the mean ± SD. n.s. no significance; *P < 0.05; **P < 0.01; ***P < 0.001 by two-tailed Student’s *t* test.

To further determine the role of miR-188 in type H vessels and bone regenerative ability, the similar bone defects stated in earlier experiments were created in young (3-month-old), middle-aged (6-month-old), and old (15-month-old) *Mir188*^*−/−*^ mice. We found more CD31^high^Emcn^high^ ECs in the bone regeneration area from the middle-aged and older *Mir188*^*−/−*^ mice than from the age-matched WT control mice (Fig. 5A, B). The bone volume was also higher in the bone regeneration area of the middle-aged and older *Mir188*^*−/−*^ mice than their WT controls as shown by micro-CT analysis (Fig. 5C–G). However, the above differences were not detected between the young *Mir188*^*−/−*^ mice and their WT controls (Fig. 5A–G).

These data suggested that miR-188 knockout alleviated the age-related type H vessel decline and weakened bone regeneration in vivo.

### miR-188-3p inhibited angiogenesis by targeting ITGβ3

**Fig. 6:**
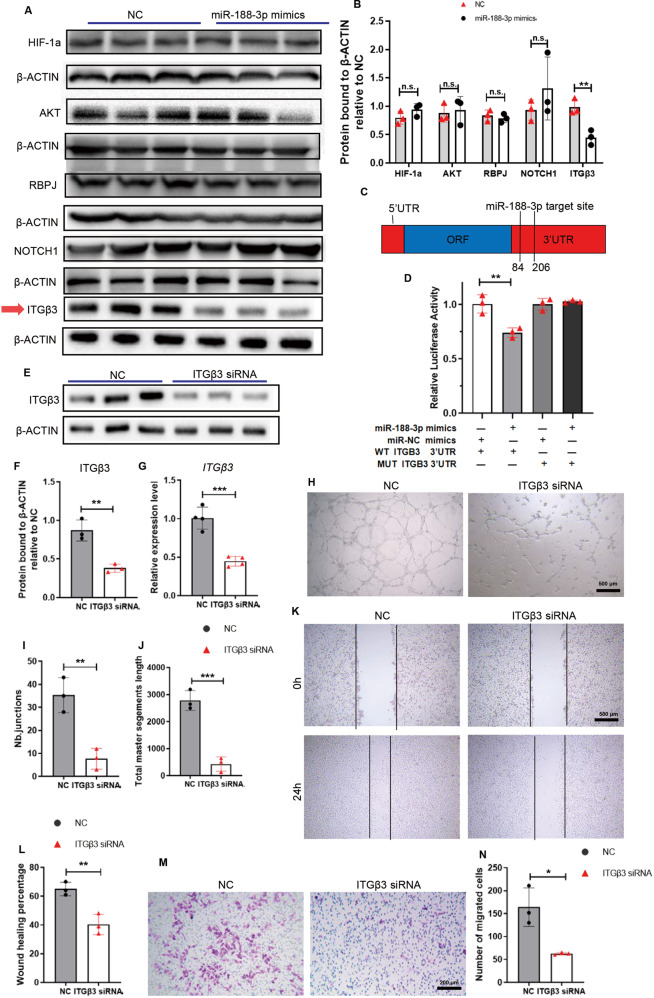
miR-188-3p inhibited angiogenesis by targeting ITGβ3. **A**, **B** Western blotting analysis of the potential and angiogenesis-related targets of miR-188-3p expression in the HMEC-1 cells transfected with miR-188-3p mimics or miRNA-NC. *n* = 3 in each group. **C** Schematic of miR-188 targeting the 3′UTR of *ITGβ3* mRNA. **D** The luciferase activity of luciferase reporter plasmids containing *ITGβ3*-WT 3′UTR and *ITGβ3*-MUT 3′UTR cotransfected with miR-188-3p mimics or miRNA-NC in HMEC-1 cells. *n* = 3 in each group. **E**, **F** Representative images of Western blotting (**E**) and quantitative analysis of ITGβ3 protein expression levels (**F**) in the HMEC-1 cells transfected with ITGβ3-siRNA or siRNA-NC. *n* = 3 in each group. **G** Quantitative analysis of *ITGβ3* mRNA expression levels in the HMEC-1 cells transfected with ITGβ3-siRNA or siRNA-NC. *n* = 4 in each group. **H**–**J** Representative images (**H**) and quantification (**I**, **J**) of tube junction numbers and total master segments length of tube formation assay from the indicated groups. Scale bar = 400 μm. *n* = 3 in each group. **K**, **L** Representative images (**K**) and wound healing percentage (**L**) of the HMECs transfected with ITGβ3-siRNA or siRNA-NC in the wound healing assays. Scale bar = 500 μm. *n* = 3 in each group. **M**, **N** Representative images (**M**) and quantification (**N**) of migrated cells from a Transwell migration assay. Scale bar = 200 μm. *n* = 3 in each group. Data are shown as the mean ± SD. n.s. no significance; *P < 0.05; **P < 0.01; ***P < 0.001 by two-tailed Student’s *t* test.

To investigate the direct targets of miR-188-3p, we predicted potential mRNA targets using online bioinformatics tools, including TargetScan [36], miRanda [37], and miRDB [16]. There are more than 4000 genes as potential targets of miR-188-3p. We chose targets that possess binding sites for miR-188-3p both in humans and mice and are also related to angiogenesis. Thus, *NOTCH*, *HIF-1a*, *AKT*, *RBPJ*, and *ITGβ3* were chosen for wet lab screening [3, 8, 38]. Western blot analysis revealed that ITGβ3, but not NOTCH, HIF-1a, AKT, or RBPJ, significantly decreased in protein levels in the miR-188-3p-treated HMEC-1 cells compared to the miR-NC-treated control cells (Fig. 6A, B). qPCR analysis showed that the mRNA level of *ITGβ3* was not affected by miR-188-3p treatment, indicating that miR-188-3p may post-transcriptionally regulate ITGβ3 (Fig. S4J). Sequence analysis revealed 1 conserved binding site of miR-188-3p in the 3′UTR of *ITGβ3* (Fig. 6C). To further verify the direct targeting of miR-188-3p on *ITGβ3*, we generated luciferase reporter constructs containing the predicted miRNA-binding site of *ITGβ3* (WT-pGL3-*ITGβ3*) or with 2 mutated nucleotides within the binding site (MUT-pGL3-*ITGβ3*). A luciferase assay showed that overexpression of miR-188-3p suppressed luciferase activity in the WT-pGL3-*ITGβ3* group but not in the MUT-pGL3-*ITGβ3* group, indicating that miR-188-3p directly targets *ITGβ3* by binding the 3’UTR of *ITGβ3* (Fig. 6D). To verify that *ITGβ3* mediates the suppressive effects of miR-188-3p on angiogenesis, we transfected HMEC-1 cells with *siRNA-ITGβ3* to knockdown *ITGβ3* and then performed tube formation, Transwell, and migration assays. Western blot analysis and qPCR analysis showed the successful knockdown of ITGβ3 in the HMEC-1 cells with *siRNA-ITGβ3* transfection (Fig. 5E–G). Intriguingly, knockdown of ITGβ3 decreased tube formation and migration of HMEC-1 cells (Fig. 5H–N).

These data indicated that miR-188-3p suppresses angiogenesis by directly targeting ITGβ3.

## Discussion

Type H vessels were shown to couple angiogenesis and osteogenesis and are thus involved in bone development and bone homeostasis [3, 4, 9]. Type H vessels decline during ageing, and the mediator and underlying mechanism remains unclear. We here report that miR-188-3p expression increases in senescent ECs and negatively regulates type H vessels during ageing. Mice with miR-188 knockout showed an ameliorated age-related type H vessel decline. Endothelial cell-specific overexpression of miR-188-3p reduced type H vessels and impaired bone regeneration, especially in aged animals. ITGβ3 was identified as the direct molecular target of miR-188-3p in regulating angiogenesis. Our study suggests that miR-188-3p, as a key mediator of angiogenesis, may be a potential target for preventing the age-related type H vessel decline and bone loss.

Type H vessels are a capillary subtype in the skeletal system with distinct morphological, molecular and functional properties. Type H vessels highly express CD31 and Emcn and are localized in metaphyseal trabecular bone subjacent to the growth plate. We found that the number of CD31^high^ Emcn^high^ type H vessels declined dramatically in 15-month-old WT mice compared to 1-month-old WT mice. However, the number of CD31^low^ Emcn^low^ type-L vessels was not dramatically affected during ageing. Thus, vessels indeed express Emcn and CD31, but not all vessels express high levels of CD31. We found that CD31^high^ Emcn^high^ type H vessels were decreased in 15-month-old WT mice. This observation is consistent with a previous report [3]. However, the underlying mechanism by which type H vessels decline during ageing has not been fully elucidated. In our previous study, we reported that miR-188-5p expression increases in BMSCs during ageing and regulates osteogenesis and adipogenesis [15]. In current study, we further elucidated the different functions between the miR-188 subtypes of miR-188-5p and miR-188-3p. We found that both miR-188-5p and miR-188-3p expression increased in ECs. Meanwhile, only miR-188-3p showed a repressive effect on angiogenesis, but not miR-188-5p. More intriguingly, we found that miR-188-3p did not affect osteogenesis and adipogenesis. Thus, although the expression of both miR-188-5p and miR-188-3p increased during ageing, miR-188-3p and miR-188-5p have different biological functions and roles in regulating angiogenesis and osteogenesis. Therefore, our finding that the mice with endothelial cell-specific overexpression of miR-188-3p had a lower bone mass and impaired bone regeneration, supported the pathological effect of the reduced type H vessels caused by miR-188-3p, but not the effect of miR-188-3p on osteogenesis.

Since type H vessels couple with osteogenesis, promoting type H vessel formation could ameliorate bone loss in ageing-, estrogen deficiency- and glucocorticoid-induced osteoporosis mouse models [4–6]. However, it is still unclear whether the decline in type H vessels contributes to decreased bone regeneration in ageing. In this study, we used two mouse models, *Mir188*^−*/*−^ mice and mice with endothelial cell-specific overexpression of miR-188-3p (AAV-TIE-miR-188-3p), to investigate whether type H vessels can also affect bone regeneration. We found that older *Mir188*^−*/*−^ mice showed increased type H vessels and improved bone regeneration. Consistently, young mice with endothelial cell-specific overexpression of miR-188-3p showed reduced type H vessels and impaired bone regeneration. These data suggest that type H vessels are tightly involved in bone regeneration and that promoting type H vessels *via* targeting miR-188-3p is a good and practical strategy to improve bone fracture healing in future study.

We also screened the molecular targets of miR-188-3p, and *ITGβ3* was identified as a direct target mediating the effect of miR-188-3p on angiogenesis. Imamaki et al. reported that ITGβ3 forms complexes with VEGFR-2, which results in enhanced VEGF-A signaling [39]. Transcripts for integrins are enriched in type H ECs, and the abundance of type H vessels in bone is controlled by endothelial ITGβ1, which suggests that the integrin family may be crucial for maintaining the normal morphological and functional properties of the type H endothelium [25]. We here found one molecular mechanism of that miR-188-3p decreases type H vessels by targeting *ITGβ3* in ECs. However, we cannot exclude the possibility that other targets of miR-188-3p are also involved in the regulation of type H vessels, which needs further studies.

In summary, we first discovered the role of miR-188-3p in regulating the age-related type H vessel decline in vivo and in vitro. Moreover, we verified that type H vessels are coupled with bone regeneration. Thus, promoting type H vessels by targeting miR-188-3p could be a potential strategy to alleviate senile bone loss and promote bone damage repair.

## Materials and methods

### Mice

The miR-188 knockout mice were produced as described previously [15]. Heterozygous miR-188 knockout mice were crossed with each other and produced homozygous miR-188 knockout mice and wild-type mice for experimental needs. For genotyping of miR-188, PCR was carried out using the following primers: forward, 5′-TCTTGGTCCGCATGTGTGTG-3′, and reverse, 5′-AGGGAGTTCAAAGGCAGCATG-3′. Endothelial cell-specific miR-188-3p-overexpressing mice were created by injecting adeno-associated virus HBAA2/9-TIE-mmu-miR-188-3p (Hanbio Biotechnology Co., Ltd., Shanghai) into the bone marrow cavity of the femur. The mice used in this study were male and on a C57BL/6J background.

The bone regeneration animal model was established as reported previously [40, 41]. We cut the skin of the anaesthetized mouse at the knee joint to expose the patellar ligament. A hole was made at the intercondylar notch of the femur by a small drill, and then, Kirschner wire, which was 0.6 mm in diameter, was placed in the femur. One week after the operation, bone samples were collected for subsequent experiments. Fourteen mice in the NC control group and 14 mice in the miR-188-3p overexpression group were used to create a bone regeneration model. After evaluation of the animal model by micro-CT analysis with the standard that an animal model with the bone regeneration area located in the metaphyseal trabecular bone subjacent to the growth plate was considered a successful model, only the data of successful animal models were included in the datasets.

All animal care protocols and experiments were reviewed and approved by the Animal Care and Use Committees of the Laboratory Animal Research Center at Xiangya Medical School of Central South University. All mice were maintained in the specific pathogen-free facility of the Laboratory Animal Research Center at Central South University.

### Endothelial progenitor cell isolation and culture

As reported previously [42, 43], under sterile conditions, we euthanized the mice and collected bone marrow from the femurs and tibias. Then, mononuclear cells were obtained by density gradient centrifugation and transferred to dishes that contained endothelial culture medium, including serum, growth factors, penicillin, and streptomycin (Lonza, Switzerland). Nonadherent cells were removed after 72 h, and adherent cells continued to be cultured.

### Induction of senescence

The human microvascular endothelial cell line HMEC-1 was cultured with MCDB131 medium (gibco, 2318307) composed of FBS (gibco, 2275120), penicillin, and streptomycin. An appropriate number of HMEC-1 cells were plated in a six-well plate. After 12 h, the original medium was changed to new medium containing H_2_O_2_, and cultivation continued for 3 days. Then, we verified senescence by β-gal staining, expression of *ink4a-p16 and* SASP factors, γ-H2AX and phosphorylated p53 according to the manufacturer’s protocol.

### Tube formation assay

As previously described [44], the Matrigel matrix was transferred to a 96-well plate, which was precooled at −20 °C and placed at 37 °C for 0.5 h to let the matrix solution solidify. A suspension of human microvascular ECs that experienced 1 h serum starvation was placed in each well, and images were captured at 4 and 8 h after incubation at 37 °C. The total length of the tubes and the number of tube branches were quantified in five randomly selected fields from each well.

### Transwell assay

Transwell assays were performed as reported previously [6]. The migration assay was carried out in Transwell 24-well plates with 8-μm pore filters. A 200 μl suspension containing 1 × 10^5^ cells that experienced 2 h of serum starvation was placed in the upper chamber. After 24 h of cultivation, the cells that adhered to the upper surface of the chamber were gently removed with cotton swabs, and the cells located at the lower surface were immersed in 4% PFA for 30 min and then stained with 0.5% crystal violet. The number of migrated cells was counted in microscope visual fields.

### BMSC isolation and culture

Bone marrow cells were collected from the femora and tibia of mice by centrifugation under sterile conditions [45]. We resuspended the pellet in α-MEM with FBS, penicillin, and streptomycin after removing the supernatant and transferred the cell suspension to a petri dish. The next day, we added PBS wash buffer to remove suspended cells and then cultured the cells with the new medium. Cells were passaged using trypsin and transferred into a six-well plate for subsequent experiments [15, 46].

### Osteogenic differentiation and adipogenic differentiation of BMSCs

BMSCs were plated in six-well plates at an appropriate density with osteogenic induction medium (α-MEM, Biological Industries) composed of FBS, penicillin, streptomycin, dexamethasone, b-glycerol phosphate and ascorbate-2-phosphate. The osteogenic induction medium was changed every 3 days. The osteogenic ability of BMSCs was detected at 7 days with qPCR analysis (*Alp*, *Runx2*, and *Sp7*) and at 21 days by Alizarin Red staining.

BMSCs were cultured in 6-well plates at an appropriate density with adipogenic induction medium (α-MEM containing FBS, 3-isobutyl-1-methylxanthine, insulin, and dexamethasone). The adipogenic induction medium was changed every 3 days. The adipogenic differentiation ability of BMSCs was detected at 3 days with qPCR analysis (*Fabp4* and *Pparg*) and 14 days by Oil Red O staining [15, 45].

### Wound healing assay

Human microvascular ECs or BMSCs were seeded in a 6-well plate. When the cells were grown to 70% confluency, cell transfection was performed. A linear wound was created by a 200-µl sterile pipette tip when the cells were grown to 100% confluency, and then, the cells were cultured in serum-free medium after cleaning with PBS. The wound healing rate was measured in each well at the 24 h timepoint.

### Transfection experiment

miR-188-3p mimics, miR-188-5p mimics, siRNA-ITGβ3 and related controls were transfected into target cells (HMEC-1 or BMSCs) using Lipofectamine 2000 according to the instructions.

### qRT-PCR analysis

According to the instructions, total RNA was extracted using TRIzol reagent and was reverse transcribed using a reverse transcription kit. The mRNA levels of the target genes were detected by qRT‐PCR using a Fluorescence Quantification PCR Kit, with β-actin as the internal control. For microRNA analysis, total RNA was reverse transcribed with a microRNA reverse transcription kit, and then, the RT-PCR product was used for qRT-PCR with the qRT-PCR kit, with U6 as an internal control. All primers were purchased from companies and were highly efficient and specific. The sequences of the primers are displayed in Table 1.

**Table 1:** List of oligonucleotide primer pairs used in the qRT-PCR analysis.

Target gene	Forward primer (5′ to 3′)	Reverse primer (5′ to 3′)
*P16*	ATCCAGGTGGGTAGAAGGTC	CCCCTGCAAACTTCGTCCT
*IL-1*	CATTGAGCCTCATGCTCTGTT	CGCTGTCTGAGCGGATGAA
*IL-6*	ACTCACCTCTTCAGAACGAATTG	CCATCTTTGGAAGGTTCAGGTTG
*IL-8*	ACTGAGAGTGATTGAGAGTGGAC	AACCCTCTGCACCCAGTTTTC
*TNF-a*	CCTCTCTCTAATCAGCCCTCTG	GAGGACCTGGGAGTAGATGAG
*Fabp4*	AAGGTGAAGAGCATCATAACCCT	TCACGCCTTTCATAACACATTCC
*Pparg*	ATGGTTGACACAGAGATGC	GAATGCGAGTGGTCTTCC
*Sp7*	ATGGCGTCCTCTCTGCTTG	TGAAAGGTCAGCGTATGGCTT
*Alp*	CCAACTCTTTTGTGCCAGAGA	GGCTACATTGGTGTTGAGCTTTT
*Runx2*	GAAATGCCTCCGCTGTTATG	AGGTGAAACTCTTGCCTCGTC
*ITGβ3*	GTGACCTGAAGGAGAATCTGC	CCGGAGTGCAATCCTCTGG
*CD31*	AACAGTGTTGACATGAAGAGCC	TGTAAAACAGCACGTCATCCTT
*EMCN*	AGCAACCAGCCGGTCTTATTC	AGCACATTCGGTACAAACCCA

### Western blot

Total protein was obtained by lysing cells with RIPA buffer containing protease inhibitor and centrifuging the cell lysate at 4 °C/10,000 rpm for 15 min. The protein concentration was detected by Coomassie assays. Total proteins were mixed with 5 × SDS‐PAGE loading buffer, boiled for 10 min and separated by SDS-PAGE followed by transmembrane. The membranes were incubated with primary antibodies against ITGβ3 (Wanleibio, WL02735), AKT (cell signaling technology, 9271s), RPBJ (abcam, ab180588), HIF-1a (novus, NB100-105), NOTCH1 (cell signaling technology, 3608S), p53 (santa cruz, sc-126), p-p53 (santa cruz, sc-51690), β-actin (proteintech, 60008-1-Ig) and GAPDH (ORIGENE, TA802519) at 4 °C overnight and with a secondary antibody for 1 h. Then, the proteins on the membranes were visualized by SuperSignal West Pico PLUS Chemiluminescent Substrate (SD251210, Thermo Fisher Scientific, Inc.). We have uploaded all the uncropped scans of blots used for quantification in the Supplementary Fig. 5.

### Flow-cytometry analysis

We crushed the metaphysis region of the femora and tibia from mice in ice-cold PBS. Bone pieces were immersed in MCDB131 medium containing 1 mg/mL type II collagenase at 37 °C for 45 min, and then, the mixed suspension was filtered with a 100 µm cell filter to obtain single-cell suspensions. The cells were blocked with 0.5% BSA for 15 min after removing red blood cells and incubated for 45 min at 4 °C with Endomucin antibody (Santa Cruz, SC-65495, 1:50) and APC-conjugated CD31 antibody (R&D Systems, FAB3628A, 1:100). The fluorescent secondary antibody was added to the deposit after washing. Finally, we obtained the data with a fluorescence-activated cell sorting (FACS) FACScan cytometer (BD Immunocytometry Systems). We analysed the CD31^high^ Emcn^high^ cell population after eliminating CD45-positive and CD11b-positive cells.

### Immunofluorescence staining

Femora were isolated from mice and incubated in 4% paraformaldehyde solution for 4 h at 4 °C. The samples were decalcified with 0.5 mol/L EDTA (pH 7.45) for 24 h or 48 h according to the age of the mice. Then, the bone samples were dehydrated with 20% sucrose and 2% polyvinylpyrrolidone (PVP) solution. Finally, the bone tissues were embedded in a mixed solution composed of 8% gelatine, 20% sucrose and 2% PVP. Bone sections (25 μm thick) were stained with primary antibodies against mouse CD31 (Abcam, ab28364) and Endomucin (Santa Cruz, V.7C7). The slides were restained with fluorescent-conjugated secondary antibodies at room temperature for 1 h. We used isotype-matched controls, monoclonal rat IgG2A (R&D Systems, 54447), under the same concentrations and conditions as negative controls [3]. The region of interest we chose to quantify the CD31^high^ Emcn^high^ vessels is a 300-µm length of metaphyseal trabecular bone immediately subjacent to the growth plate. Immunofluorescence staining was analysed at high resolution with a Zeiss laser scanning confocal microscope. All quantifications were performed with ImageJ software on high-resolution confocal images, specifically, the ratio of the CD31^high^ Emcn^high^ area (i.e., yellow area) to the total area of interest.

### Histochemistry and immunocytochemistry staining

Histochemistry and immunocytochemistry staining were carried out as reported previously. Femora were isolated from mice and incubated in 4% paraformaldehyde solution for 24 h at 4 °C. Then, the tissues were immersed in ice-cold 10% EDTA (pH 7.4) on a shaker for decalcification, and the buffer was changed every other day until 21 days. Finally, the samples were embedded in paraffin after gradient dehydration. Five-micrometer-thick sections were needed for the next experiments. TRAP and HE staining was performed with a standard protocol. For immunocytochemistry staining, the slides of bone tissues were incubated with primary antibodies against Ocn (Takara Bio, Inc., M137) at 4 °C overnight on a shaker. A horseradish peroxidase streptavidin detection system (Dako) was applied to test the immunoactivity, and we finally stained the nucleus with haematoxylin (Sigma-Aldrich).

### μCT analysis

We conducted µCT analysis using high-resolution μCT (Skyscan 1172, Bruker microCT, Kontich, Belgium). The parameters of the scanner were a voltage of 63 kV, a current of 156 μA, and a high resolution of 15 μm per pixel. We used image reconstruction software (NRecon, version 1.6, Bioz, Inc, Palo Alto, CA, USA), 3-dimensional model visualization software (μCT Volume, version 2.0, Bruker microCT), and data analysis software (CT Analyser, version 1.9, Bruker microCT) to analyse the trabecular bone. The parameters of trabecular bone (Tb. BV/TV, Tb. Th, TB. N, TB. SP) were measured.

### Statistics

The data are shown as the mean ± SD; All data were normally distributed. Multiple groups were compared with one-way ANOVA. Two groups were compared with unpaired two-tailed Student’s *t* test or Welch’s *t* test. Differences were considered significant when *P* < 0.05. Sample sizes were selected on the basis of previous experiments.

## Supplementary information

Figure S1

Figure S2

Figure S3

Figure S4

Figure S5

aj-checklist

## Data Availability

All data is available from the corresponding author upon reasonable request.
