# Supplementary figures and images for: miR-188-3p targets skeletal endothelium coupling of angiogenesis and osteogenesis during ageing
Source: Cell Death Dis. 2022 May 25;13(5):494. doi: 10.1038/s41419-022-04902-w (PMC9130327; doi:10.1038/s41419-022-04902-w)

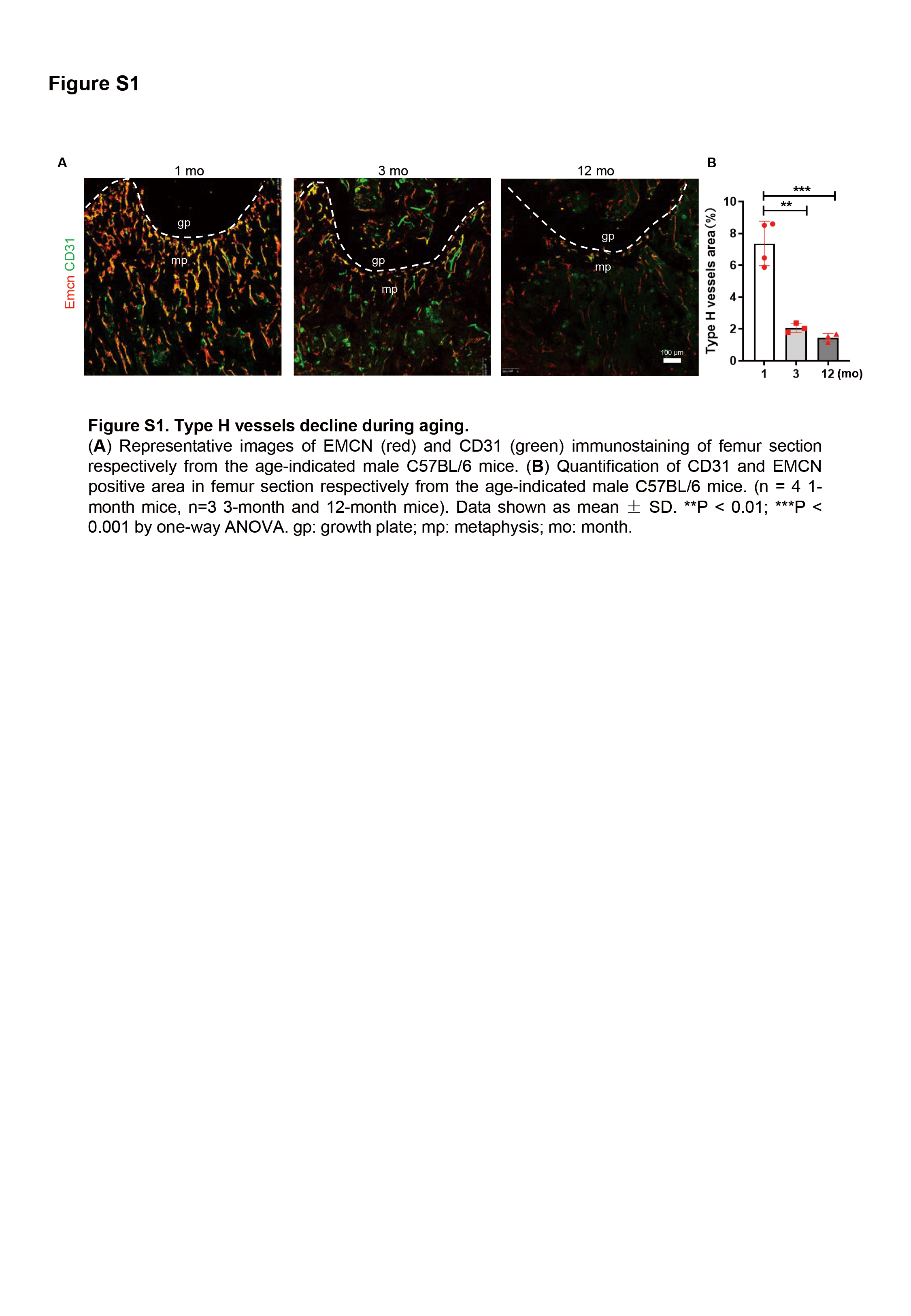

Supplement: Supplementary file 1 — Figure S1 [file 41419_2022_4902_MOESM1_ESM.jpg]

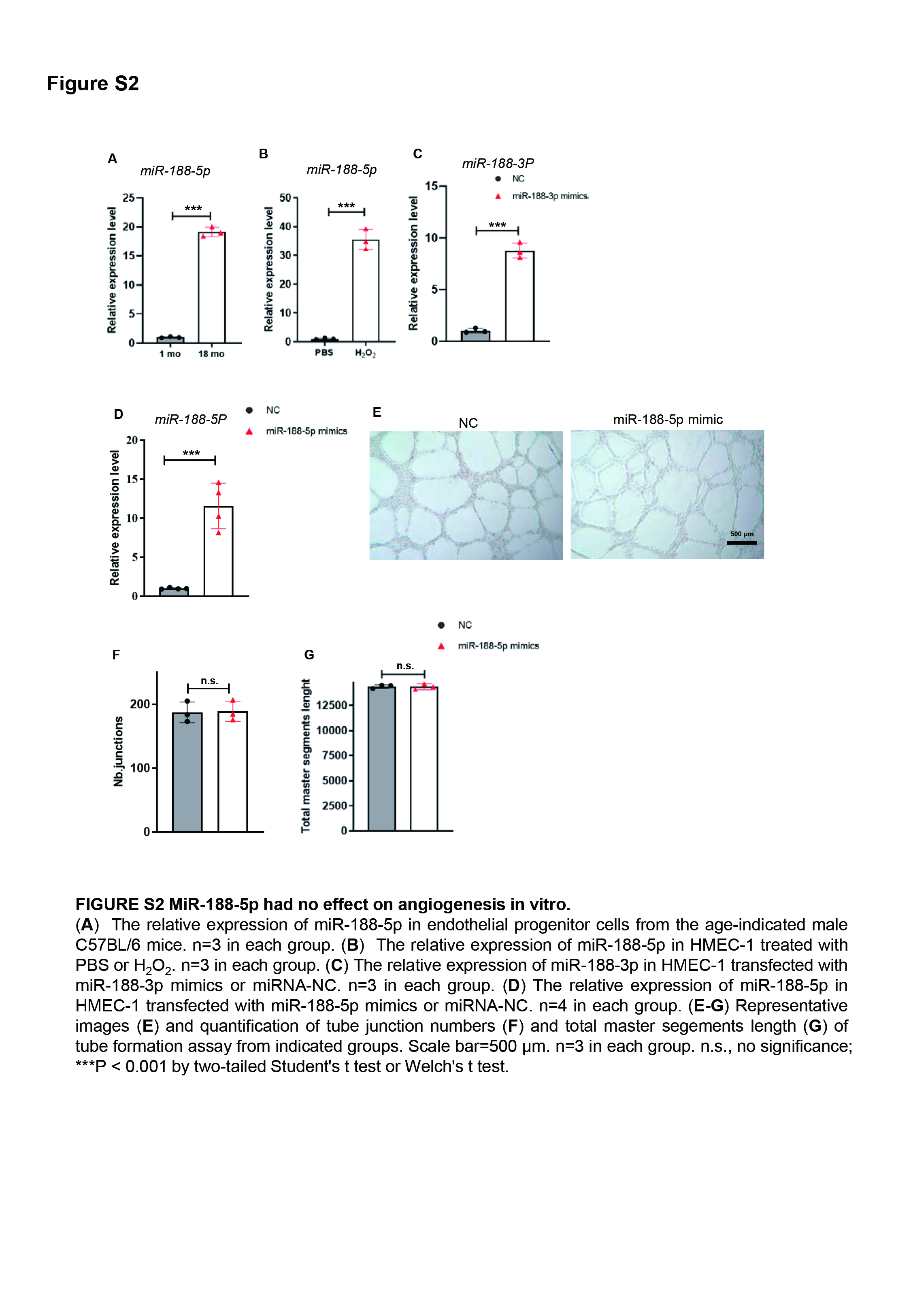

Supplement: Supplementary file 2 — Figure S2 [file 41419_2022_4902_MOESM2_ESM.jpg]

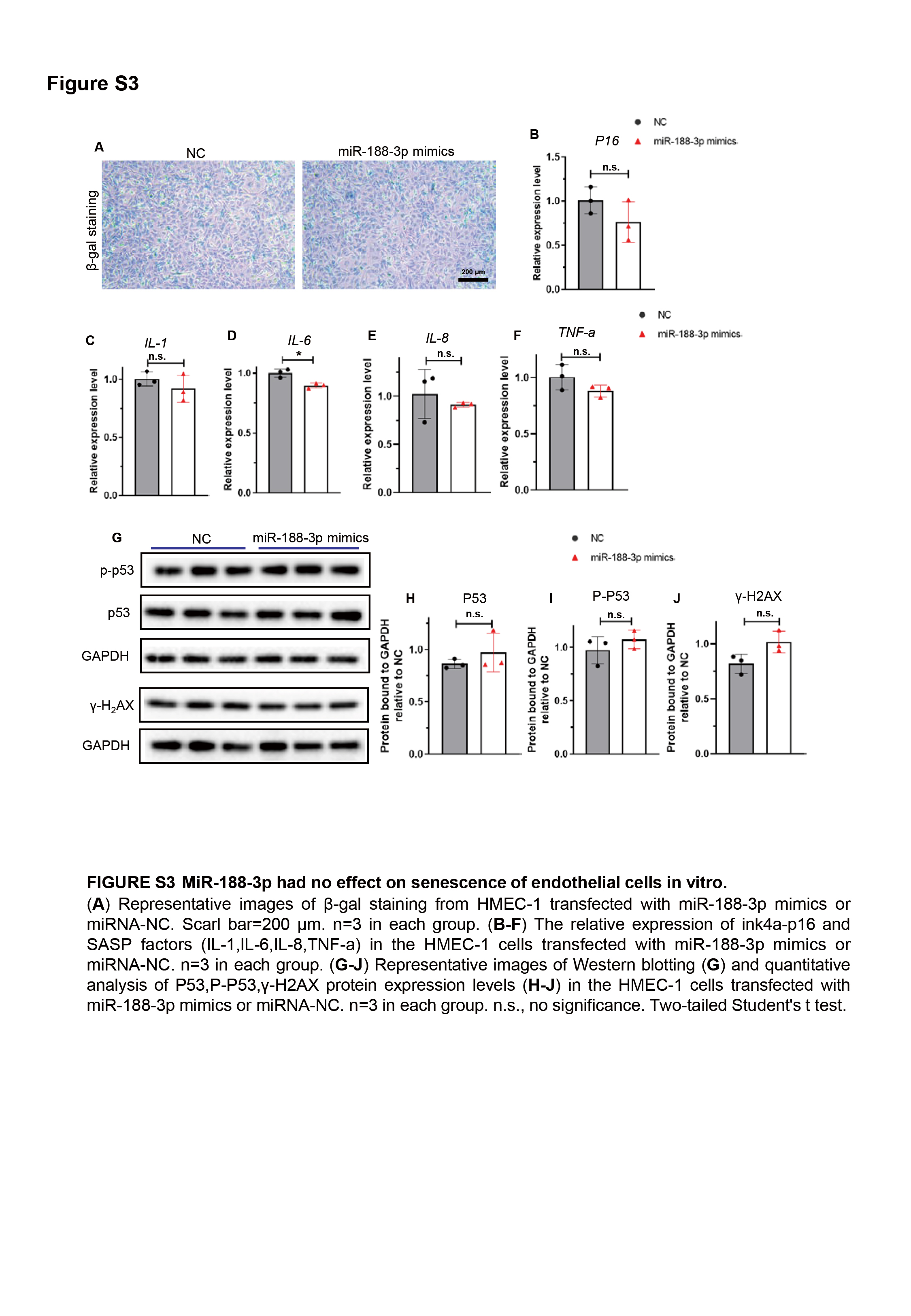

Supplement: Supplementary file 3 — Figure S3 [file 41419_2022_4902_MOESM3_ESM.jpg]

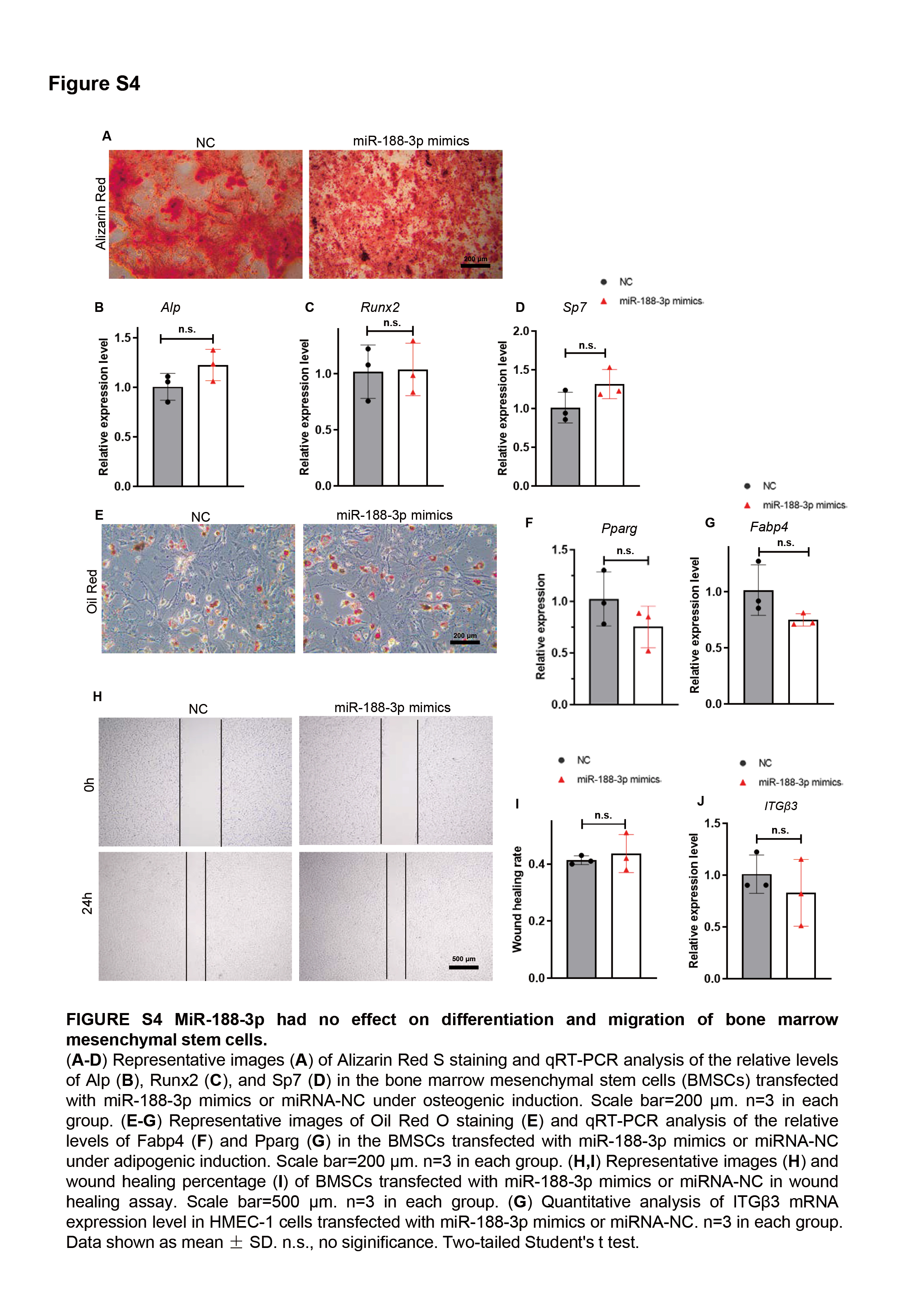

Supplement: Supplementary file 4 — Figure S4 [file 41419_2022_4902_MOESM4_ESM.jpg]

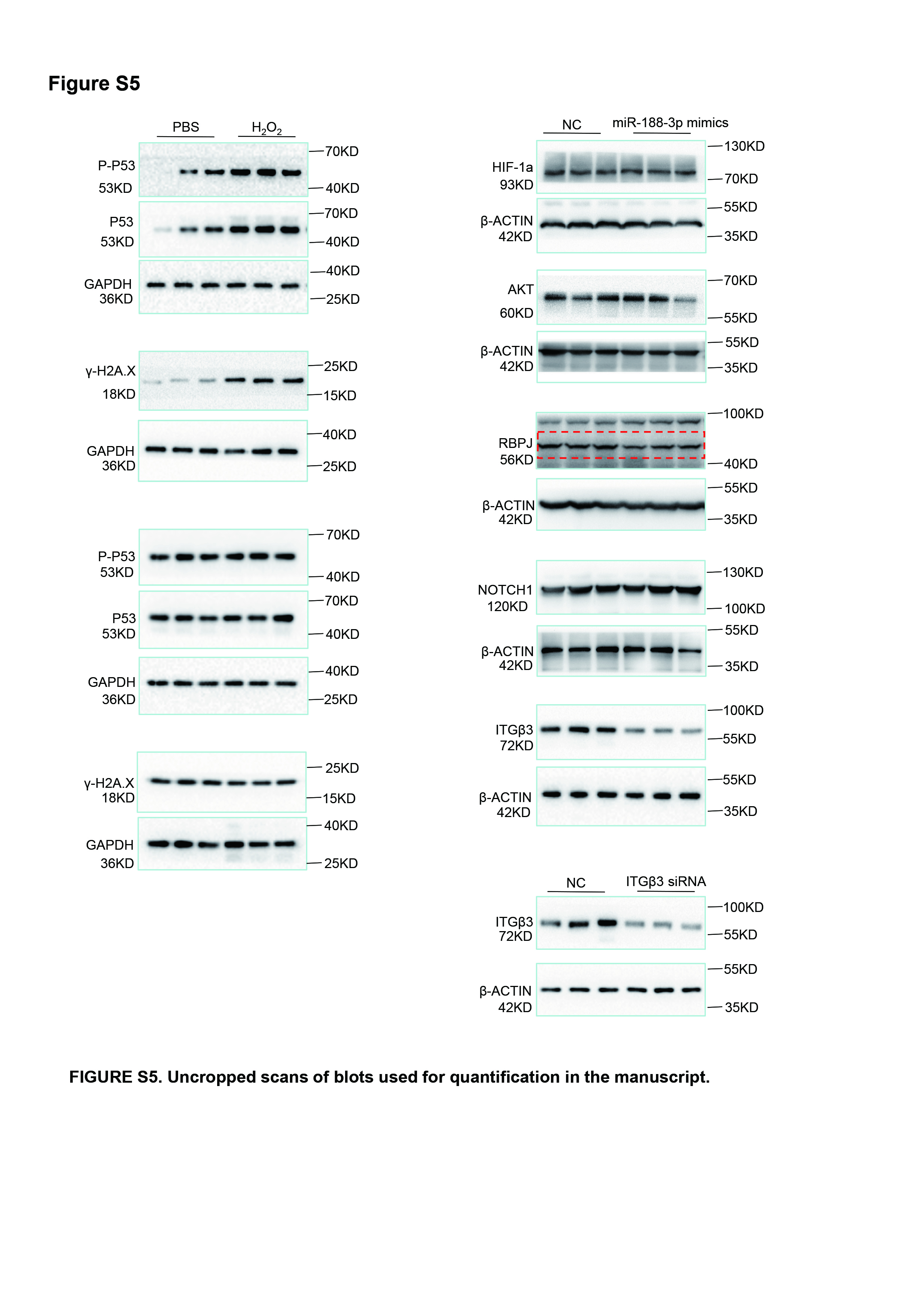

Supplement: Supplementary file 5 — Figure S5 [file 41419_2022_4902_MOESM5_ESM.jpg]
